# Retraction: Lee, D.-S. et al. The Aminopyridinol Derivative BJ-1201 Protects Murine Hippocampal Cells against Glutamate-Induced Neurotoxicity via Heme Oxygenase-1. *Molecules* 2016, *21*, 594.

**DOI:** 10.3390/molecules21111463

**Published:** 2016-11-07

**Authors:** Dong-Sung Lee, Tae-Gyu Nam, Byeong-Seon Jeong, Gil-Saeng Jeong

**Affiliations:** 1 College of Pharmacy, Chosun University, Dong-gu, Gwangju 61452, Korea; dslee2771@chosun.ac.kr; 2 Department of Pharmacy, Hanyang University, Ansan 15588, Korea; tnam@hanyang.ac.kr; 3 College of Pharmacy, Yeungnam University, Gyeongsan 38541, Korea; 4 College of Pharmacy, Keimyung University, Daegu 42601, Korea

As the authors of the title paper [1], it is with great regret that we inform the readership of *Molecules* that we have asked the journal’s publisher MDPI to retract our paper from the scientific literature. Our reason for this action is that we made a mistake inserting a previous experiment result in Figure 5a. This result was published in 2014 [2]. We were not able to find the original data that should have been used for the figure. We also considered generating a new data set for Figure 5a, but we no longer had any compound BJ-1201, which takes a long time to synthesize. In addition, Figure 5a in Nrf-2 translocation, is one of the key results to confirm the neuroprotective mechanism by BJ-1201. This paper was part of a Special Issue of *Molecules* “Neuroprotection Mediated by Natural Products and Their Chemical Derivatives”, and we wish to apologize to Prof. Dr. Peter Koulen, the Guest Editor, to MDPI and of course to the wider scientific community for any inconvenience this action may cause.
